# Frequency-pattern functional tomography of magnetoencephalography data allows new approach to the study of human brain organization

**DOI:** 10.3389/fncir.2014.00043

**Published:** 2014-04-29

**Authors:** Rodolfo R. Llinás, Mikhail N. Ustinin

**Affiliations:** ^1^Department of Neuroscience and Physiology, New York University School of MedicineNew York, NY, USA; ^2^Institute of Mathematical Problems of Biology, RAS, PushchinoMoscow Region, Russia

**Keywords:** multichannel signal analysis, frequency coherence, extraction of partial spectra, time series reconstruction, functional localization

## Abstract

A method based on a set of new theorems for the analysis of multichannel time series is described, based on precise Fourier transform and coherence analysis of the restored signals from a detailed set of frequency components. Magnetic field recordings of spontaneous and evoked activity by means of magnetic encephalography demonstrated that multichannel precise Fourier spectrum contains a very large set of harmonics with high coherence. The inverse problem can be solved with great precision based on coherent harmonics, so the technique is a promising platform of general analysis in brain imaging. The analysis method makes it possible to reconstruct sites and timing of electrical activity generated by both spontaneous and evoked brain function at different depths in the brain in the millisecond time range.

## Introduction

Modern scientific studies are performed by means of new powerful equipment, generating large amounts of detailed data. Magnetic encephalography (MEG) provides an example of a foremost biological technology, comparable with the most sophisticated physical devices. Magnetic encephalographs register magnetic field in hundreds of channels with sampling frequency up to several thousand Hertz. Typical 5 min experiment on the 275 channel device with sampling rate 1200 Hz, provides 100 million field values, so the problem of data analysis appears a pressing challenge in the MEG technique. Many approaches are used to solve various scientific and diagnostic problems of encephalography. Fourier analysis in many implementations can be called the oldest and the most popular of methods used for the brain data analysis (Dietsch, 1932; Jansen et al., 1981; Halliday et al., 1995). Through the whole history of this method it was connected with difficulties of calculations, so the development of the Fast Fourier Transform (FFT) (Cooley and Tukey, 1965) dramatically advanced the application of the Fourier analysis in many fields, including brain research (Miyashita et al., 2003). Regularization of the FFT was performed in multitaper method (Thomson, 1982; Percival and Walden, 1993), implemented in the studies of the evoked and spontaneous activity (Llinás et al., 1999; Mitra and Pesaran, 1999). In the quantitative electric and magnetic encephalography studies, trying to study patterns of the Fourier coefficients, rough spectral analysis is combined with statistical analysis of coherence between channels or independent components (Tauscher et al., 1998; Weiss and Rappelsberger, 2000; Jarvis and Mitra, 2001; Makeig et al., 2002; Delorme and Makeig, 2004; Garcia-Rill et al., 2008; Muthuraman et al., 2010). Usually in applications of the Fourier analysis to brain studies the spectra are calculated in short (<10 s) time windows, based on the well-known property of instability of the brain processes (Llinás, 2001).

Typically brain studies register activity in many channels simultaneously for protracted time periods (up to tens of minutes in hundreds of channels). Those registered data are usually processed with two important methodological weaknesses: First weakness is that in time dependence analysis the methods are applied, which were developed for the solitary time series, multichannel recordings are implemented mainly to attempt inverse problem solutions. The second weakness lies in the usage of short time windows (less than 10 s), decreasing the resolution of the Fourier transform.

These procedural limitations relate to the fact that only descriptive conclusions can be drown from such studies. Thus, it is often stated, that the particular pathology or evoked activity results in the spectral changes of some kind or another. Such qualitative approach diminishes a detailed analysis quite drastically, while it can be useful from a general diagnostic perspective of view or to study some general cognitive features. At the same time that approach to data analysis loses practically all experimental information, and narrows encephalography to general sets of particular observations.

Recently the method of precise frequency-pattern analysis to decompose complex systems into functionally invariant entities was proposed (Llinás and Ustinin, 2012). The method makes it possible to address general spectra to the partial spectra of stable functional entities and to restore their time series. The method is based on the complete utilization of the long time series, while the multichannel nature of the data is also completely taken into account, making it possible to implement detailed reconstruction of neuronal circuit activity.

## Methods

In multichannel recording of the brain activity, such as those from MEG, a magnetic field event is recorded by many channels at discrete time moments, providing sets of experimental vectors {**b**_*k*_}, where *k* is number of channel, the *l*-th component of vector **b**_*k*_ = b_*k*_ (*l*) is the result of field measurement at the time moment *t_l_, l* = 1,…,*L*.

The first step in the method proposed is the interpolation of the experimental data in every channel, providing the continuous function

(1) $${\tilde{B}}_{k}(t)=F(b_{k},t),$$

where *F* is a function, corresponding to the particular method of interpolation (Boyd, 2001). The linear and spline interpolation methods are used in our study with satisfactory results.

After interpolation the multichannel recorded signal is described by the set of functions {$\tilde{B}$_*k*_ (*t*)}, where *t* ∈ [0, *T*], *T* = *t_L_* − *t*_1_, *T* is the whole time of measurement, *k* = 1,…, *K*, *K* is maximal number of channel.

The multichannel precise Fourier transform calculates a set of spectra for interpolated functions {$\tilde{B}$_*k*_(*t*)}:

(2) $$\begin{array}{l} a_{0k}=\frac{2}{T}\int_{0}^{T}{\tilde{B}}_{k}(t)dt,a_{nk}=\frac{2}{T}\int_{0}^{T}{\tilde{B}}_{k}(t)\cos\text{​}(2\pi \nu_{n}t)dt,\\ \;b_{nk}=\frac{2}{T}\int_{0}^{T}{\tilde{B}}_{k}(t)\sin\text{​}(2\pi \nu_{n}t)dt, \end{array}$$

where *a*_*0k*_, *a_nk_*, *b_nk_* are Fourier coefficients for the frequency ν_*n*_ in the channel number *k*, and *n* = 1,…, *N*, *N* = ν_max_*T*, where ν_max_ is the highest desirable frequency.

The term “Precise” is used in three different senses here and is achieved by three distinct steps:

1. Precise calculation of the Fourier integrals. Gaussian quadrature formulas are used to calculate integrals on any interval [0, *T*], in the registration scale.
2. Building all spectra for the total registration time *T*, as opposed to methods using moving or fractional window. The step in frequency is equal to $\Delta \nu =\nu_{n}-\nu_{n\;-\;1}=\frac{1}{T}$, thus frequency resolution is determined by the recording time.
3. Tuning of the frequency grid by cutting the interval of integration *T* to build the optimal approximation of the frequency selected. Tuning can be performed by little changes of the integration time *T*.

The method can be implemented without interpolation, if Fourier integrals can be calculated with a required accuracy on the experimental set of time points *t_l_*, *l* = 1,…, *L* and if tuning of the frequency grid is not necessary.

This precise transform leads to an accurate and reversible representation of time data in the frequency domain for each channel. As for the space domain, “space” is determined by the simultaneous registration by multiple channels having different positions with respect to the source. That is, if an accurate representation of time series for all channels is used, spatial characteristics of the signal can also be determined accurately.

Given a precise multichannel spectra it is possible to perform the inverse Fourier transform using:

(3) $$\begin{array}{l} B_{k}(t)=\frac{a_{0k}}{2}+\sum_{n=1}^{N}a_{nk}\cos\text{​}(2\pi \nu_{n}t)+\sum_{n=1}^{N}b_{nk}\sin\text{​}(2\pi \nu_{n}t),\\ \;\nu_{n}=\frac{n}{T},\;N=\nu_{\max}T \end{array}$$

where *a_0k_*, *a_nk_*, *b_nk_* are Fourier coefficients, found in (2).

This formula can also be written as

(4) $$B_{k}(t)=\frac{a_{0k}}{2}+\sum_{n=1}^{N}\rho_{nk}\sin\text{​}(2\pi \nu_{n}t+\varphi_{nk}),\;\nu_{n}=\frac{n}{T},\;N=\nu_{\max}T,$$

where $\rho_{nk}=\sqrt{a_{nk}^{2}+b_{nk}^{2}},\varphi_{nk}=\text{atan}2(a_{nk},b_{nk})$.

The transform (3) or (4) allows the possibility of implementing precise filtering, including or eliminating any selected set of frequencies when restoring the signal.

We propose to study the detailed frequency structure of the brain, restoring multichannel signal at every frequency and analyzing the patterns obtained.

The multichannel signal is restored at particular frequency in all channels:

(5) $$B_{nk}(t)=\rho_{nk}\sin\text{​}(2\pi \nu_{n}t+\varphi_{nk}),$$

where *t*∈ [0,*T*_ν_*n*__], *k* = 1,…,*K* and $T_{\nu_{n}}=\frac{1}{\nu_{n}}$ is the period of this frequency.

The summary instantaneous power produced by all channels at the frequency ν_*n*_ will be:

$$p_{n}(t)=\sum_{k=1}^{K}B_{nk}^{2}(t).$$

The proximity of phases φ_*nk*_ in different channels can be characterized by the value of empirical one-frequency coherence:

(6) $$C_{1f}=1-\frac{\underset{t\in \left[0,T_{\nu_{n}}\right]}{\min}(p_{n}(t))}{\underset{t\in \left[0,T_{\nu_{n}}\right]}{\max}(p_{n}(t))},$$

where min and max are calculated at the period *T_ν_n__*. Possible values of the coherence lay between 0 and 1: *C*_1*f*_ ∈ (0,1]. The physical sense of *C*_1*f*_ follows from formula (5). If all channels have equal phases φ_*nk*_ = ν_*n*_ at the frequency ν_*n*_, then *C*_1*f*_ is equal to 1. If phases in different channels are distributed uniformly and amplitudes are equal, then *C*_1*f*_ approaches to zero when maximal number of channels *K* is growing.

The pattern of magnetic field at the time moment *t* is determined by relation between values of the induction in different channels and by their average energy. Relative values make it possible to determine the spatial structure of the source from the inverse problem solution, and this structure is the same for the same relative values of the channels. If φ_*nk*_ = φ_*n*_, then formula (5) can be written as

(7) $$B_{nk}(t)=\rho_{nk}\sin\text{​}\left(2\pi \nu_{n}t+\varphi_{n}\right)={\overset{⌢}{\rho }}_{nk}\rho_{n}\sin\text{​}(2\pi \nu_{n}t+\varphi_{n}),$$

where $\rho_{n}=\sqrt{\sum_{k\;=\;1}^{K}\rho_{nk}^{2}}$ and ${\overset{⌢}{\rho }}_{nk}=\frac{\rho_{nk}}{\rho_{n}}$.

From the formula (7) it follows that relative values of channels $\overset{⌢}{\rho }$_*nk*_ are independent of time. The inverse problem solutions, determined by the normalized pattern $\overset{⌢}{\rho }$_*nk*_, have the same spatial structures for any moment of the restored time. The amplitude of the source is determined by ρ_*n*_ sin (2πν*_n_t* + φ_*n*_)—common for all channels, meaning that this source is oscillating as a whole at the frequency ν_*n*_.

The following theorems have been proved.

**Coherence Theorem 1**. The equality of phases in all channels is a necessary and sufficient condition for normalized pattern invariance through reconstructed time.
**Conclusion 1**. If for particular frequency phases are equal in all channels, then the spatial structure of the source at this frequency can be found by the solution of inverse problem for the pattern $\overset{⌢}{\rho }$_*nk*_.
**Coherence Theorem 2**. The equality of phases in all channels is necessary and sufficient condition for the equality of the empirical one-frequency coherence to 1, *C*_1_*f* = 1. This theorem provides a directly calculable feature to estimate the proximity of phases in all channels at any frequency ν_*n*_.
**Coherence Theorem 3**. The time course of the magnetic field source, having arbitrary spatial structure, can be restored from the partial Fourier spectrum. This partial spectrum consists of the frequencies with *C*_1*f*_ equal or close to 1, having the same normalized pattern. Spatial structure of the source can be found from this pattern.

Consider the equivalent current dipole (ECD), characterized with two vectors: **r**_0_ —is the location of the dipole and **Q**—is a dipolar moment. The model of ECD in spherical conductor (Sarvas, 1987) is used to calculate the magnetic induction registered by sensor, having the location **r** and direction **n**:

(8) $$B({\text{r}}_{0},Q)=\frac{\mu_{0}}{4\pi F^{2}}\left(\left(F\text{​}\left(Q\times r_{0}\right)-\left(Q\times r_{0},r\right)\nabla F\right),n\right),$$

where *F* = *a(ar* + *r*^2^ − (**r_0_, r**), ∇*F* = (*a*^2^ *r*^−1^ + *a*^−1^ (**a**, **r**) + 2*a* + 2r) **r** − (*a* + 2*r* + *a*^−1^ (**a**, **r**)) **r**_0_,

$$a=r-r_{0},a=\left|a\right|,r=\left|r\right|,\left|n\right|=1,\mu_{0}=4\pi \cdot 10^{-7}.$$

It can be shown, that magnetic induction depends linearly on the dipole moment and can be written as:

(9) $$B\left(r_{0},\;Q\right)=\left(\frac{\mu_{0}}{4\pi F^{2}}\left(\left(r_{0}\times n\right)F-\left(\nabla F,n\right)\left(r_{0}\times r\right)\right),Q\right)=\left(K,Q\right),$$

where $K=\frac{\mu_{0}}{4\pi F^{2}}\left(F\left(r_{0}\times n\right)-\left(\nabla F,\;n\right)\left(r_{0}\times r\right)\right)$.

From the formula (9) and the principle of superposition it follows, that the induction, measured by the sensor number *k* from *J* dipoles can be written as (Hamalainen et al., 1993):

(10) $${\tilde{B}}_{k}=\sum_{j=1}^{J}\left(K_{kj},Q_{j}\right).$$

Consider a coherent system, consisting of J dipolar sources, having similar time dependencies: ***Q**_j_* = *c_j_* **$\overset{⌢}{Q}$**_*j*_ *Q(t)*, where *Q(t)* is a function of time, common for all dipoles, *c_j_* gives the force of dipole number *j*, **$\overset{⌢}{Q}$**_*j*_ is a unitary vector, giving the direction of this dipole.

The formula (10) now can be written as

(11) $${\tilde{B}}_{k}(t)=Q(t)\sum_{j=1}^{J}P_{kj}c_{j},$$

where *P_kj_* =(**K***_kj_*,**$\hat{Q}$**_*j*_). The lead field matrix element *P_kj_* is given by the sensing character of the sensor number *k* in relation to the source number *j*. After the summation

(12) $${\tilde{P}}_{k}=\sum_{j=1}^{J}P_{kj}c_{j},$$

(13) $${\tilde{B}}_{k}(t)=Q(t){\tilde{P}}_{k}.$$

After the precise Fourier transform it follows from the formula (13) that for every frequency the restored signal in the *k*-th channel will be

(14) $$B_{nk}(t)={\tilde{P}}_{k}\rho_{n}\sin\text{​}(2\pi \nu_{n}t+\varphi_{n}),$$

where ρ_*n*_ sin (2πν*_n_t* + φ_*n*_) = *Q_n_(t)* is the *n*-th Fourier component of the function *Q(t)*.

Formula (14) can be written in the form (7):

(15) $$B_{nk}(t)=P{\hat{P}}_{k}\rho_{n}\sin\text{​}(2\pi \nu_{n}t+\varphi_{n}),$$

where $P=\sqrt{\sum_{k\;=\;1}^{K}{\tilde{P}}_{k}^{2}\;}\text{ and }{\hat{P}}_{k}=\frac{{\tilde{P}}_{k}}{P}$.

The instantaneous power will be: *p_n_(t)* = *P*^2^ρ^2^_*n*_ sin^2^ (2πν*_n_t* + φ _*n*_), and it has minimum = 0 in the period, so *C*_1*f*_ is equal to 1 for every frequency of the function *Q(t)*. From (15) it also follows, that normalized patterns at all frequencies of the function *Q(t)* will be identical. This makes possible to extract the partial spectrum, corresponding to this source, from the full multichannel spectrum. This is implemented by selecting frequencies with high coherence, having equal or similar normalized patterns. From this partial spectrum the time course *Q(t)* can be restored by the inverse Fourier transform.

These theoretical considerations are the foundation for the reconstruction of time courses of static functional entities (neural circuits, or sources), performing detailed frequency analysis and studying the similarity of the patterns with high coherence. Also spatial structure of the sources at separate frequencies with high coherence can be restored, leading to the total decomposition of the brain activity.

The algorithm of mass precise frequency-pattern analysis can be summarized as:

1. Precise Fourier Transform of the multichannel signal.
2. Inverse Fourier Transform—restoration of the signal at each frequency.
3. If the coherence at the particular frequency is close to 1, then use the pattern and frequency as elementary coherent oscillation.
4. If the restored signal consists of several phase-shifted coherent oscillations, then extract those oscillations.
  After the fourth step the initial multichannel signal will be represented as a sum of elementary coherent oscillations. Each elementary oscillation has distinct frequency, constant pattern and being produced by the functional entity having constant spatial structure. The set of elementary coherent oscillations is unique for the subject and for the particular experiment.
5. Split the spectrum to the assembly of partial spectra, based on the extraction of frequencies with similar normalized patterns.
6. Solve inverse problem for normalized patterns in order to determine spatial structure of the functionally invariant entities.
7. Restore the time courses from partial spectra of functionally invariant entities.

After the seventh step all brain activity recorded is decomposed to the set of activities of functional entities, with known spatial structures and time courses.

The method can be called “Frequency-Pattern Functional Tomography,” because it reveals the structure and function of the brain under study in the particular experiment.

## Experimental results

The method proposed makes it possible to perform detailed study of the brain structure and function by means of multichannel measurements, such as magnetic or electric encephalography. Note, that Theorems 1–3 make sense only if the coherence of multichannel oscillations is high at the frequency under study. In order to estimate the applicability of the method to the real data, MEG experimental data sets for 19 persons (control subjects and patients) were processed. Nine data sets were obtained with a 148-channel magnetometer Magnes 2500 WH, and 10 data sets were obtained with a 275-channel gradiometer. Both systems were installed in the Bellevue Hospital in the Center for Neuromagnetism of New York University School of Medicine. These experiments were performed during research studies of human spontaneous and evoked activity, including control subjects and patients with various disorders (Llinás et al., 1999; Ustinin et al., 2010). The NYU Institutional Review Board and Bellevue Hospital Research Protocol Review Group approved the study and an informed written consent was obtained from all subjects before the MEG recording.

As an example of the Precise Fourier analysis, consider the auditory experiment JG03_01. The MEG data were obtained with a 275-channel synthetic third order gradiometer (VSM MedTech LTD) at Bellevue Hospital in the Center for Neuromagnetism at the Department of Neuroscience and Physiology of the New York University School of Medicine. The auditory stimulus was a 2 ms click, presented at 14 Hz into the left ear of a healthy subject, and the recording was implemented.

**Figure 1:**
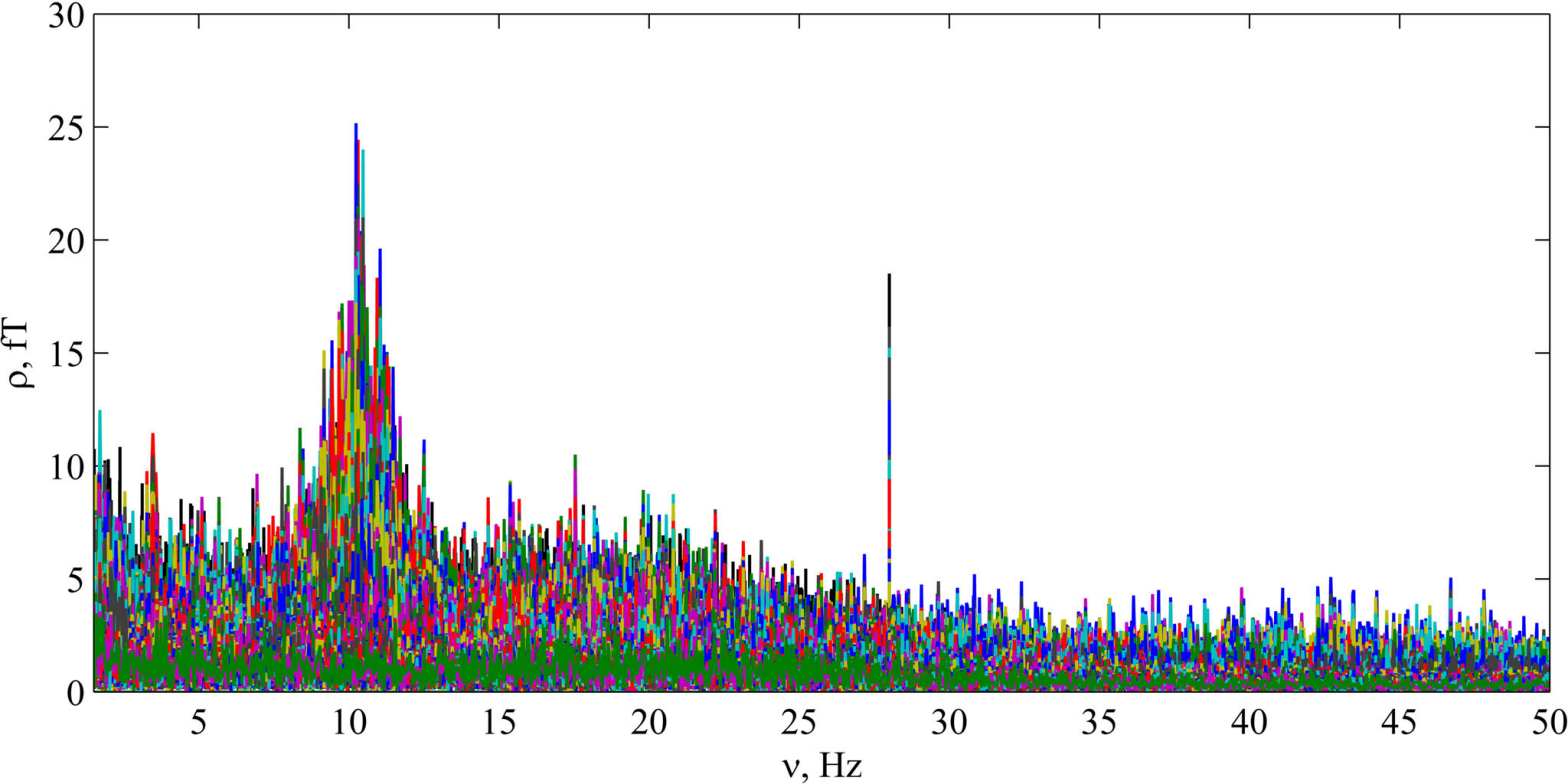
**Precise multichannel Fourier spectrum for the auditory recording JG03_01**. Multiple peaks corresponding to alpha activity can be seen near 10 Hz. Also the second harmonic of the stimulus frequency is well noticeable at 28 Hz.

**Figure 2:**
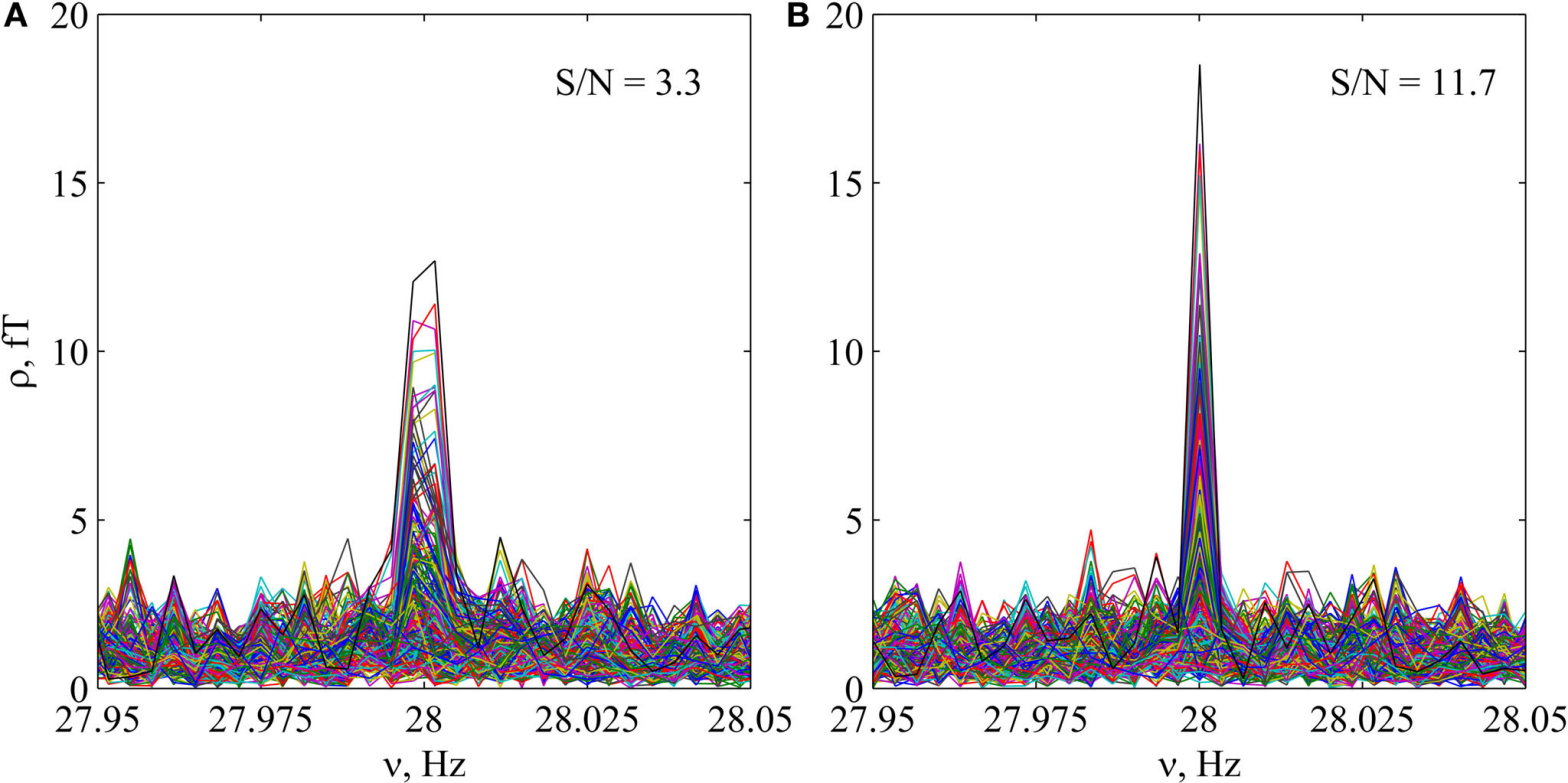
**Tuning of the precise multichannel Fourier spectrum for the stimulus frequency in auditory MEG experiment JG03_01. (A)** Multichannel spectrum close to the second harmonic of the stimulus, calculated using *T* = 300 s. **(B)** The same spectrum, calculated using *T* = 300 − 0.017 s. It can be concluded, that a negligible lost of information significantly increases the signal to noise ratio (S/N), leading to much higher coherence. More precise solution of the inverse problem can be obtained after such optimization.

**Figure 3:**
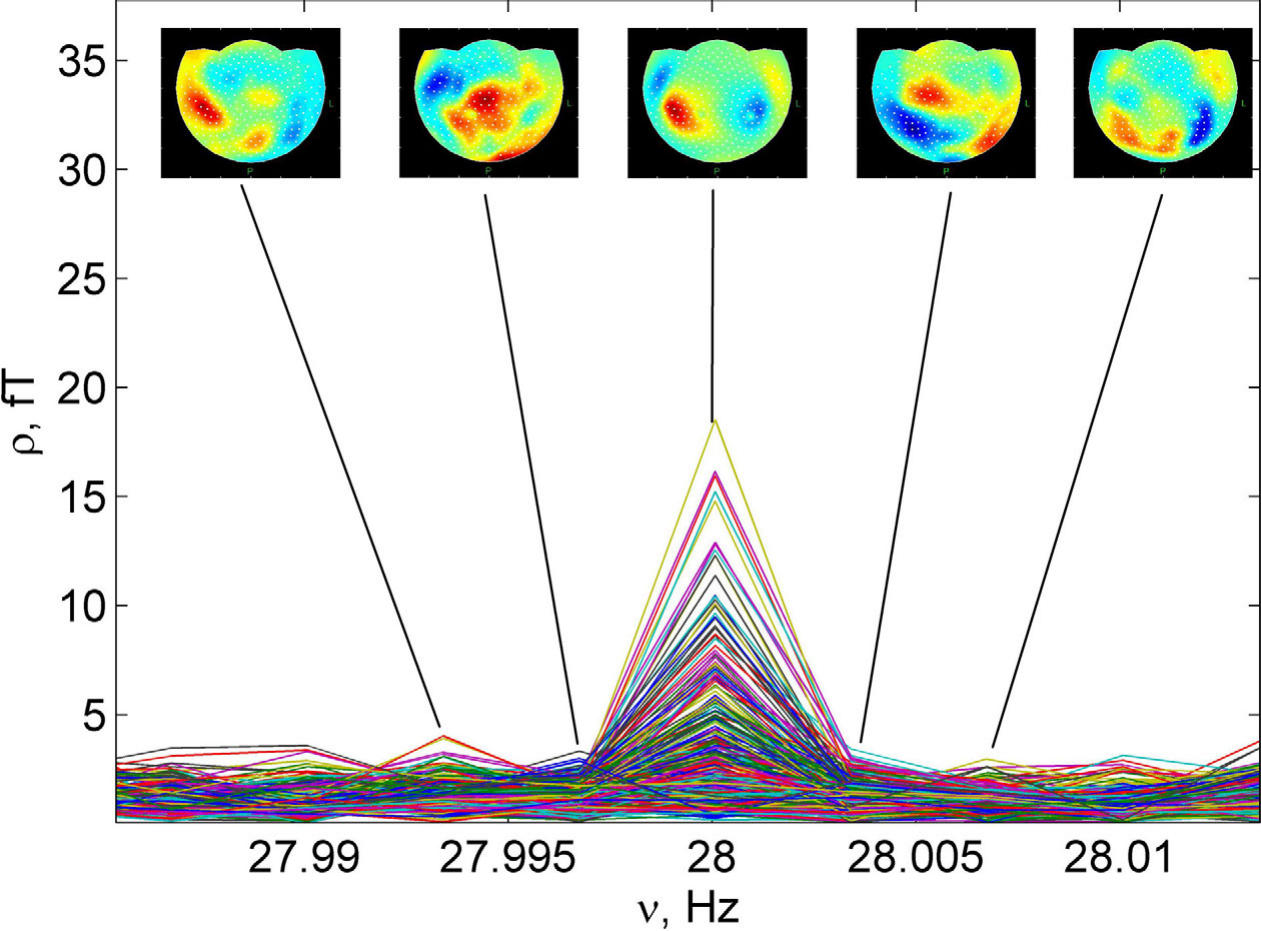
**Detail of the Figure 2B, close to the maximum of spectrum at 28 Hz, (the 2nd harmonic of the stimulus)**. Five normalized patterns $\overset{⌢}{\rho }$_*nk*_ (7) of the restored MEG, corresponding to neighboring frequencies, are shown.

**Figure 4:**
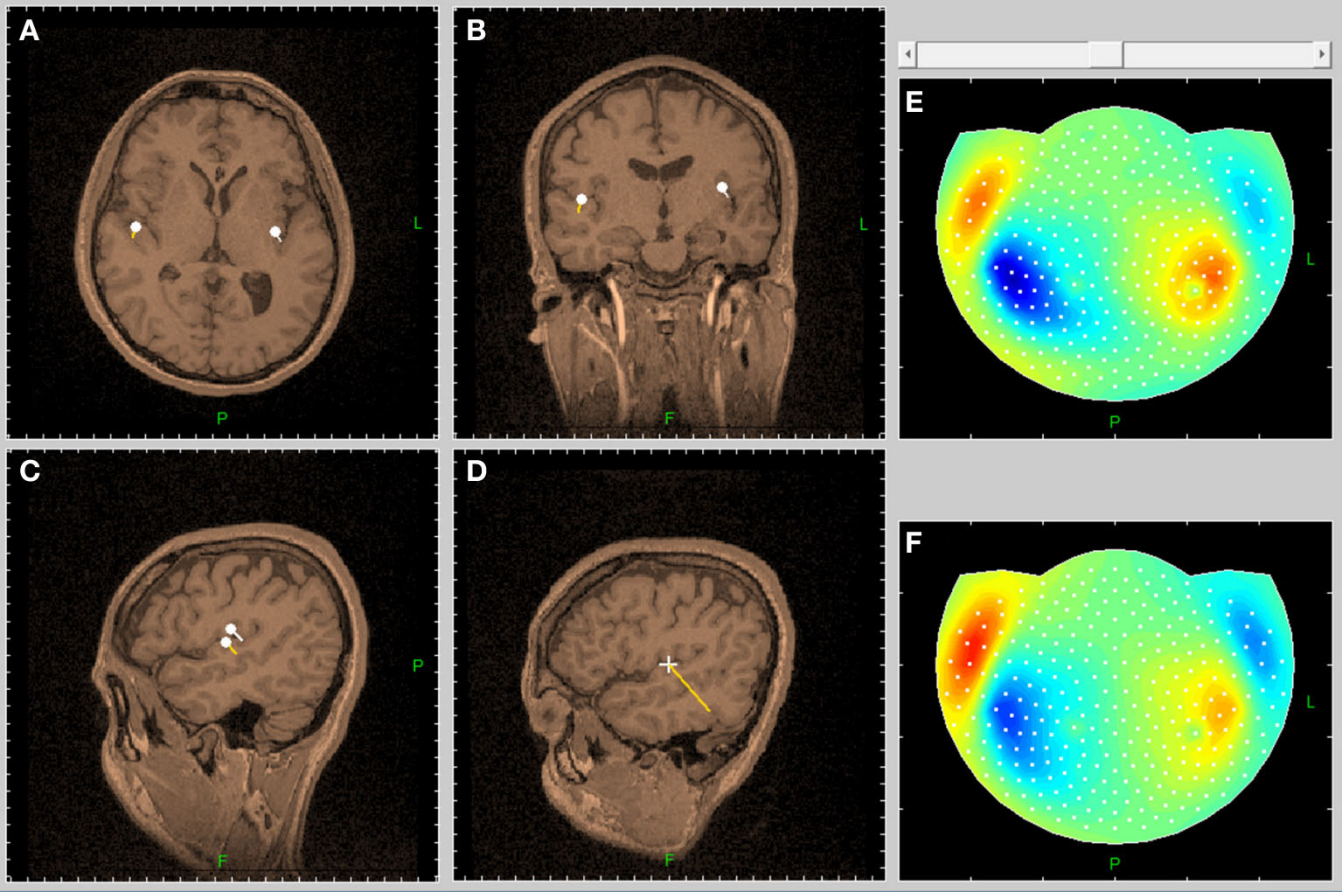
**The inverse problem solution reveals the spatial structure of the functional entity, producing the pattern of auditory response (Figure 3, middle pattern)**. This structure is well described by the two-dipole model. **(A–D)** Show the sections of the tomogram, going through the right dipole. **(E)** Shows the map of experimental magnetic field. **(F)** Shows the map of the field, produced by the inverse problem solution.

The precise multichannel Fourier spectrum was calculated, using the whole registration time (~300 s) and sampling rate 1200 Hz. Figure 1 illustrates the multichannel spectrum in the frequency range 1.5–50 Hz. This spectrum was calculated with $\Delta \nu =\nu_{n}-\nu_{n-1}=\frac{1}{300}$ Hz and contains 15,000 frequencies in 275 channels, plotted simultaneously. It can be concluded, that this plot gives only general impression about the spectrum, and detailed quantitative analysis is necessary. Such precise analysis is illustrated in Figures 2–4.

The tuning of the frequency grid was done by minor cutting the time of registration T to build an optimal approximation of the basic stimulus frequency and its harmonics (Figure 2). This tuning increases the signal to noise ratio by the order of magnitude in power, giving the possibility to solve the inverse problem with high precision.

A large scale in frequency was utilized which produces a precise spectrum obtained that is fractioned into separate frequencies with different patterns of Fourier amplitudes.

The fragment of the spectrum in narrow frequency band shown in Figure 3, is close to the second harmonic of the stimulus. The normalized patterns $\overset{⌢}{\rho }$_*nk*_ (7) of the restored magnetic fields are also presented for five neighboring frequencies. Also illustrated in Figure 3 is the extraction of the response to stimulus from spontaneous activity. The frequency peak at the second harmonic of the stimulus produces normalized pattern, corresponding to auditory cortical activity. The frequency to analyze was selected from the precise Fourier transform of the stimulus time course. Following the inverse Fourier transform of this frequency, highly coherent oscillation presents second harmonic of the brain response to auditory stimulus. The structure of the functional entity, generating this response, can be found from the inverse problem solution (Figure 4).

**Figure 5:**
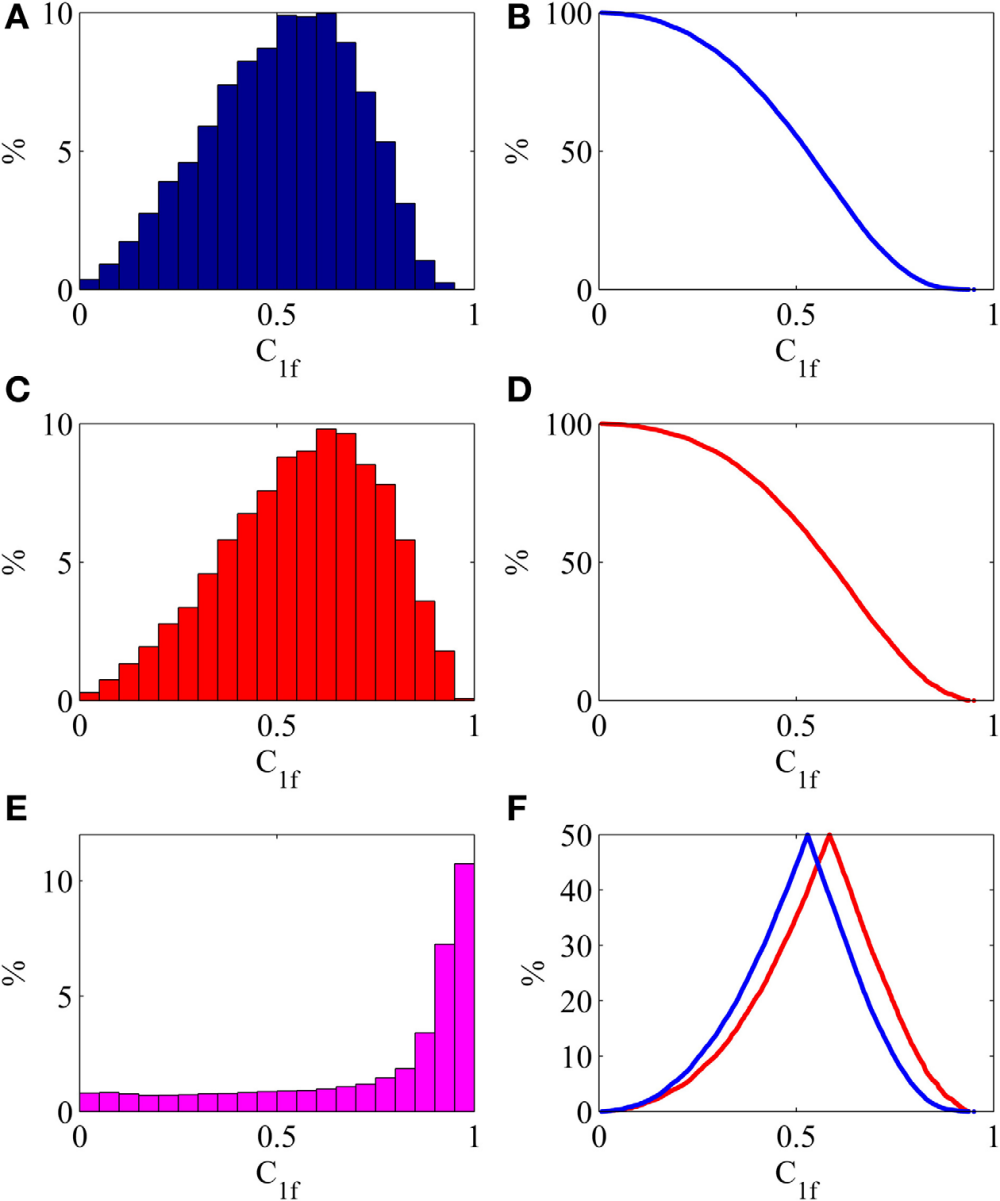
**Statistical properties of the restored oscillations**. *C*_1*f*_ —one frequency empirical coherence (6). **(A)** Shows relative number of frequencies, falling in the 5%-bin of coherence. **(B)** Shows share of frequencies, having coherence greater than chosen value. **(C)** Shows relative summary energy of frequencies, falling in the 5%-bin of coherence. **(D)** Shows share of common energy, produced by frequencies, having coherence greater than chosen value. **(E)** Shows distribution of the relative one-frequency energy, falling in the 5%-bin of coherence. For example, average energy, produced by one frequency with coherence >0.95, is 11 times greater than average energy, produced by one frequency. **(F)** Shows folded cumulated distribution functions, calculated from **(B)** blue and **(D)** red.

In order to study general properties of the restored multichannel Fourier components, the statistical analysis of the coherence distribution was performed (Figure 5). For every frequency, the inverse Fourier transform (5) was performed and the empirical coherence (6) was calculated. Also the summary energy for every frequency was calculated.

It was found that, in this experiment, mean coherence was 0.51. Moreover 4.42% of frequencies have coherence greater than 0.8 and 0.25% of frequencies have coherence greater than 0.9. (Figures 5E,F). This illustrates the fact, that the highest amplitude frequencies have the greatest coherence.

For example, 4.42% of all frequencies, with coherence greater than 0.8, represent 11.26% of all energy, while 0.25% of frequencies with coherence greater than 0.9 produce 1.87% of total energy. Statistical properties of the restored multichannel one-frequency oscillations are illustrated by Figure 5. The conclusion is that there are many frequencies with high coherence, providing the direct information about the functional entities, generating those frequencies.

Precise multichannel Fourier spectra were calculated for all 19 data sets, using (2). For all frequencies of the every spectrum the multichannel inverse Fourier transform (5) was performed, the empirical coherence *C*_1*f*_ (6) was calculated, and the analysis described in Figures 1–5 was performed. The results were similar to those described in Figures 1–5, with the average coherence 0.523.

It was found, that:

1. Precise multichannel spectra of the MEG data parcel to oscillations with different patterns.
2. Both in spontaneous and evoked experimental spectra there are many frequencies with coherence, close to 1.
3. MEG sensory evoked activity allow attaining data with high coherence at the selected stimulus frequencies, which disclose the brain functional structure generating particular responses.

The conclusion is that Theorems 1–3 can be applied to the experimental data in multichannel magnetic and electric encephalography, giving a direct nonparametric method to reveal functional entities in the human brain, oscillating as whole systems.

In some cases, when applying the above method to real data, the value of empirical coherence is less than 1.

This may occur for various reasons, namely:

1. Non-correlated noise, produced by the system under study, including sensors noise. This noise can be reduced through experimental design, for example, increasing the time of measurement *T*.
2. Activity of different non-correlated sources, falling at the same frequency band $\nu_{n}\pm \frac{1}{2T}$.

This is typical for most methods utilizing Fourier analysis, especially when moving or fractional windows are utilized. The precise Fourier transform can address the issue imaging activity from different non-correlated sources by either increasing the recording time *T* or/and by tuning of the frequency grid to an exact frequency.

3. Activity of several coherent sources, shifted in phase, having exactly the same frequency ν_*n*_ and also falling at the same frequency band $\nu_{n}\pm \frac{1}{2T}$.

In the third case, simultaneous activity of coherent sources with different spatial structures can indicate functional connectivity. To divide different coherent processes from the restored multichannel signal independent component analysis of patterns activity can be used, or those processes can be separated directly. This procedure leads to the extraction of patterns, produced by several different sources with high coherence at the same frequency. It can reveal the physiological dependence of the activity sources at this frequency.

## Discussion

A very large set of multichannel MEG recordings have been processed using precise frequency-pattern analysis. Following this procedure it has been found, that for many frequencies, empirical coherence (formula 6) is close to 1. As follows from the Coherence Theorem 1, normalized pattern for the particular frequency with high coherence is close to invariant. This means that the functional entity, producing this pattern, is oscillating as a single entity at this frequency. Thus, the activity of the elementary subsystem of the brain can be extracted and its spatial structure determined from the inverse problem solution. In the particular case of magneto-encephalography this spatial structure is very robust as no arbitrary parameters were used to extract the pattern. Also in case of the MEG the inverse problem solution does not need subject parameters, except the head shape (Hamalainen et al., 1993).

Given the above the technique described is capable of addressing the structure of the brain as a set of coherent functional entities.

Note, that this method can also be applied in electroencephalography. In the case of EEG the pattern will be also extracted correctly, but the solution of the inverse problem will involve complicated brain parameters, such as the spatial distribution of conductivities. Those parameters are usually unknown, adding the uncertainty to the inverse problem solution (Fuchs et al., 2001; Hallez et al., 2005; Plis et al., 2007; Barnes et al., 2008).

Finally, our results indicate that precise spectra are sui generis every subject, in particular concerning spontaneous activity. We propose this new analysis paradigm for brain research, based on the calculation of precise spectra and on their storage for future reference concerning the development of pathological conditions, among other uses. The general number of functional entities in the particular experiment can be estimated as 5–10 thousand. This number in the order of magnitude is close to the number of categories, introduced to describe cognitive processes in Huth et al. (2012). Functional entities, revealed by the method proposed (Llinás and Ustinin, 2012), correspond to emergent functions of neural circuits (Alivisatos et al., 2012) and the study of these entities can be the important component of the starting Brain Activity Map project.

### Conflict of interest statement

The authors declare that the research was conducted in the absence of any commercial or financial relationships that could be construed as a potential conflict of interest.
